# 

**Published:** 1892-12

**Authors:** 


**Married in New York**, Thursday, December 8th, inst., at 8:30 p, m., Dr. William Wayne Ashhurst to Ellen Eyre Gaillard, daughter of Mrs. Mary E. Gaillard, and the late Prof. E. S. Gaillard. The JOURNAL acknowledges the courtesy of an invitation, and extends its best wishes for the happiness and prosperity of the young couple.

